# Supplementary material for: WGS Revealed Novel BBS5 Pathogenic Variants, Missed by WES, Causing Ciliary Structure and Function Defects
Source: Int J Mol Sci. 2023 May 13;24(10):8729. doi: 10.3390/ijms24108729 (PMC10218572; doi:10.3390/ijms24108729)
Supplement: Supplementary file 1 [file ijms-24-08729-s001.zip › ijms-2349422-supplementary.pdf]

SUPPLEMENTARY DATA

BBS panel sequencing

Next-generation sequencing of the patient's sample was performed on the Ion Torrent PGM (Thermo Fisher Scientific) according to the manufacturer's protocols. DNA libraries were constructed using the HaloPlex Target Enrichment system (Agilent Technologies, version D.5). The design targeted 30 genes of which 17 were BBS genes (*BBS1-BBS17*) for a total of 10,156 amplicons (134.04 kbp). The libraries were barcoded using HaloPlex ION Barcodes (Agilent Technologies) and then pooled by four samples. Emulsion PCR was performed on the Ion One Touch 2 system (Life Technologies) and the emulsion PCR products were enriched on the One Touch 2 Enrichment System using the Ion PGM Template OT2-200 kit (Life Technologies). Ion sphere particles (ISP) were enriched using the E/S module, charged on one Ion PGM 318 v2 chips and sequenced with an Ion Torrent PGM in a 200-bp configuration run. With an ISP loading of 73%, 481 Mb were produced in total out of 2,951,659 reads, with a median length of 166 bp, of which 130.6 Mb could be used for our patient (795,763 reads, median length of 164 bp). Sequencing data were analyzed by the Torrent Suite Software v4.4.2 with alignment to the reference human genome (GRCh37/hg19) (788,342 mapped reads, 48.61% on target) and base calling (SNV/indel). Variant annotation and ranking was performed as for the WGS methods. No specific CNV calling was achieved but depth of coverage comparison was manually checked using the Integrative Genomics Viewer (IGV) software [1].

Whole-Exome Sequencing

Whole exome sequencing (WES) was performed by the IGBMC Microarray and Sequencing platform. Genomic DNA (2 mg) was sheared to obtain a mean fragment size of 150 bp using the Covaris E210 (KBioscience, Herts, United Kingdom) followed by library preparation using the Agilent SureSelect XT Human all exon V6 (Agilent Technologies, Santa Clara, CA, United States PN G7530-90000, protocol B4). Sequencing was performed on an Illumina HiSeq 2500 (Illumina, San Diego, CA, United States) to generate 100-bp paired-end reads following the manufacturer's protocols. Bioinformatics Analysis Image analysis and base calling were performed using CASAVA v1.8.2 (Illumina), and the 181,329,380 reads were mapped to the reference human genome (GRCh37/hg19) using BWA v0.7.5a [2] leading to 97.26% of the bases covered at least by 20x. GATK UG v3.2-2 was used to call SNV and indel variations [3]. Annotation and ranking of SNV and indel were performed by VaRank 1.4.3 [4] in combination with the Alamut Batch software 1.11 (Interactive Biosoftware, Rouen, France). Very stringent filtering criteria were applied to filter out non-pathogenic variants (Supplementary Table S1): (i) variants represented with an allele frequency of more than 1% in public variation databases including the 1000Genomes [5], the gnomAD database [6], the DGV database [7] or our internal database, (ii) variants in 5' and 3' UTR, downstream, upstream or intronic locations and synonymous without pathogenic prediction of local splice effect, (iii) variants not in the ciliary genes [8]. Structural variants were predicted using CANOES, including mobile element insertion using Mobster [9] and annotated by AnnotSV 2.3 [10]. The analysis was focused on compound heterozygous and homozygous variants consistent with a recessive mode of transmission. Each candidate variations were also checked using the Integrative Genomics Viewer (IGV) software [1].

Copy number variants calling

Copy number variants have been called using 7 different tools either on the WES data with CNVkit 0.9.9 [11], on the WGS data using CNVpytor 1.2.1 [12], Delly 1.1.5 [13], Lumpy 0.2.13 [14], Smoove 0.2.6 (https://github.com/brentp/smoove) based on Lumpy [14], or on both using CANOES v2 [15], Manta 1.6.0 [16] and SoftSV 1.4.2 [17]. Detection results and type of methods are summarized in Supplementary Table S5.

BBS5 Multiple Sequence Alignment and structure

Protein sequence alignment was built based on the protein sequences extracted from the metazoan orthologous group from the eggNOG database (4.5) [18] including the sequence from the 3D structure of ARL6/BBS3 (PDB: 6VBU, chain A) [19]. The alignment was visualized in Jalview (2.11.0) [20] and the 3D structure in PyMOL (1.3) [21].

			SV	
	**SNV**	**indel**	**CANOES**	**Mobster**
Total number of variants	94,460	15,592	10	229
After exclusion of variants with an allele frequency >1% (gnomAD, 1000G, internal exome database or DGV)	10,178	1,635	6	229
After exclusion of SNV/indel found at the homozygous state in gnomAD	4,669	695		
After exclusion of SNV/indel found at the homozygous state in our internal exome database	1,486	261		
After exclusion of SNV/indel in 5' and 3'UTR, downstream, upstream, intron and synonymous locations without local splice effect prediction	520	88		
After exclusion of missense without SIFT, PPH2 or PhastCons deleterious effect	456	88		
SV overlapping a morbid gene (OMIM)			0	57

Supplementary Table S1. Summary of the whole exome sequencing results. SNV: single nucleotide variation, indel: gain or loss of up to 50 nucleotides at a single locus, SV: structural variation. Exclusion of SV with a DGV frequency >1% is done only with studies of more than 1000 individuals.

		SV			
	**SNV/Indel**	**CANOES**	**SoftSV**	**Lumpy**	**Mobster**
Total number of variants	4,851,744	10	15,518	14,441	1,472
After exclusion of variant with an allele frequency >1% (gnomAD)	468,570	5	13,882	10,720	1,401
After exclusion of SNV/indel found at the homozygous state in gnomAD	240,450				
After exclusion of SNV/indel in 5' and 3'UTR, downstream, upstream, intron and synonymous locations without local splice effect prediction	837				
After exclusion of missense without SIFT, PPH2 or PhastCons deleterious effect	18				
SV overlapping a morbid gene (OMIM)		0	1348	980	116

Supplementary Table S2. Summary of the whole genome sequencing results. SNV: single nucleotide variation, indel: gain or loss of up to *50* nucleotides at a single locus, SV: structural variation. A SV with a DGV frequency >1% is excluded only with studies of more than 1000 individuals.

Application	Gene name (HGVS)	Exon	Forward (5'-3')	Reverse (5'-3')	Size (bp)
Duplex 1	*BBS5*	F1 and R1	GGCACCAGGATTCTCATACC	TGGGTCACTTGAAGTCAGGA	389/6309
	PSMC3	7 to 8	GGCGTGTCCCTGAGTTAGAG	AGGAACCACAATTTAGCACAA	603
Duplex 2	*BBS5*	F2 and R2	TGTACACTCACGTTCCTTTGTC	CAGAGAAGCAGGCTTACAACAG	663/6580
	DISC1	10	ACCCTGTACTTGGAGGTATGTG	ACCCCTGCCCTCCAATATTTGT	925
cDNA	*BBS5*	6-7 to 8	TCCAGTGATCAGGGCAATTT	TGAGAGCTTTCTATGACAAGAGC	167
	BBS5	1 to 3	ATGCCAAAGAGTGCCAGAGA	TCCGTTTCGACCTGTCCG	153
	BBS5	5-6 to 6-7	TGGCAGTACACGAGCTTATGA	TGATCAGGGCAATTTAGGAACC	164
	BBS5	9 to 9-10	GTGGTGGATATGTTCTTGGCT	CTGGGGCTTTTCTTCCATCTC	140
	GLI1	7 to 9	CTGCAGCCAGGAATTTGACT	CGAGGCGTGAGTATGACTTCC	224
	GLI2	2 to 3	AAGCAAGAAGCCAAAAGTGG	TGGTACCTTCCTTCCTGGTG	188
	GLI3	2 to 3	GGCATTTTTGGTCGAAGAGA	GGACATTCTGTGGCTGCATA	238
	SUFU	2-3 to 3	ACAGAGTCCATGAGTTTACAGG	CGTATCGTGCCAAGCCCT	138
	PTCH1	2-3 to 3	GTGGAAGTTGGAGGACGAGT	AGTCCAGGTGTTGTAGGAGC	154
	SMO	2 to 3	TGCCCAAGTGTGAGAATGAC	TACCAGCTCTTGGGGTTGTC	229
	GAPDH	4-5 to 6	GGAGCGAGATCCCTCCAAAAT	GGCTGTTGTCATACTTCTCATGG	197
	HPRT	1-2 to 2-3	CCTGGCGTCGTGATTAGTGAT	AGACGTTCAGTCCTGTCCATAA	131

Supplementary Table S3. List of primers used in this study. Two duplex PCR have been setup with 2 primers set and different control genes (i.e. *PSMC3* and *DISC1*). Size of the PCR product for the duplex depends on the presence or not of the deletion.

Application	Antibody	Species	Dilution	Reference
**Western blot**	Anti-β-Tubulin	Mouse	1/2500	TUB-2A2, Euromedex
	Anti-BBS5	Rabbit	1/500	14569-1-AP, Proteintech
	Anti-mouse (HRP linked) II^{ary}	Horse	1/2000	7076, Cell signaling
	Anti-rabbit (HRP linked) II^{ary}	Donkey	1/2000	7074, Cell signaling
**Immunofluorescence**	Anti-ARL13B	Mouse	1/500	Sc-515784, Santa cruz biotechnology
	Anti-SMO	Mouse	1/500	Sc-166685, Santa cruz biotechnology
	Anti-γ-Tubulin	Rabbit	1/500	PA5-34815, Invitrogen
	Anti-mouse (Alexa Fluor™ 488) II^{ary}	Goat	1/500	A11001, Invitrogen
	Anti-rabbit (Alexa Fluor™ 594) II^{ary}	Donkey	1/500	A21207, Invitrogen

Supplementary Table S4. List of antibodies used in this study.

Program	Version	Method	Analysis	Detected	Nb Events	Samples
**CANOES**	v2	Read depth	WES	No	4	11
CANOES	v2	Read depth	WES	Yes	8	38
CNVkit	0.9.9	Read depth	WES	No	76	38
Manta	1.6.0	Paired end/Split read	WES	No	17	38
SoftSV	1.4.2	Read depth/Split read	WES	No	2,784	38
Delly	1.1.5	Paired end/Split read	WGS	Yes	11,447	18
**CANOES**	v2	Read depth	WGS	No	10	18
CNVpytor	1.2.1	Read depth	WGS	No	578	18
**Lumpy**	0.2.13	Paired end/Split read	WGS	Yes	14,441	18
Manta	1.6.0	Paired end/Split read	WGS	Yes	11,953	18
Smoove	0.2.6	Paired end/Split read	WGS	Yes	7,195	18
**SoftSV**	1.4.2	Read depth/Split read	WGS	Yes	15,718	18

Supplementary Table S5. Summary of bioinformatics CNV calling on either the WES or the WGS from AII.2. Programs used for the default pipeline are highlighted in bold. Number of samples have been indicated but are relevant only regarding the read depth methods using a comparative approach including the other samples in the run.

***BBS5* variant**	**SIFT-indel**	**DDIG**	**Provean**
c.550_552dup, p.Asn184dup	Damaging (0.894)	Disease causing (83.8)	Deleterious (-9.466)

Supplementary Table S6. Prediction of the BBS5 AA duplication. The indel inframe effect was assessed using SIFT [22], DDIG [23] and Provean [24].

HGVS (cDNA)	HGVS (protein)	HGMD	Type	Reference
c.1A>T	p.Met1?	DM	Start loss	Muller (2010) Hum Genet
c.2T>A	p.Met1?	DM	Start loss	Harville (2010) J Med Genet
c.149T>G	p.Leu50Arg	DM	Missense	Chen (2011) Invest Ophthalmol Vis Sci
c.148C>A	p.Leu50Ile	DM?	Missense	Wang (2014) Hum Genet
c.158C>T	p.Thr53Ile	DM	Missense	Abu-Safieh (2012) Eur J Hum Genet
c.166A>G	p.Arg56Gly	DM	Missense	Muller (2010) Hum Genet
c.214G>A	p.Gly72Ser	DM	Missense	Hjortshoj (2008) Am J Med Genet A
c.412C>T	p.Arg138Cys	DM	Missense	Carss (2017) Am J Hum Genet
c.413G>A	p.Arg138His	DM	Missense	Redin (2012) J Med Genet
c.532G>A	p.Gly178Arg	DM	Missense	Shaheen (2016) Genome Biol
c.547A>G	p.Ths183Ala	DM	Missense	Hjortshoj (2008) Am J Med Genet A
c.551A>G	p.Asn184Ser	DM?	Missense	Li (2004) Cell, Carss (2017) Am J Hum Genet
c.584A>G	p.Asp195Gly	DM	Missense	Lindstrand (2016) Am J Hum Genet
c.620G>A	p.Arg207His	DM?	Missense	Li (2004) Cell
c.790G>A	p.Gly264Arg	DM	Missense	Weisschuh (2016) PLoS One
c.889G>A	p.Asp297Asn	DM?	Missense	Feuillan (2011) J Clin Endocrinol Metab
c.265C>T	p.Arg89*	DM	Nonsense	Hirano (2015) PLoS One
c.425T>G	p.Leu142*	DM	Nonsense	Chandrasekar (2018) Indian J Med Res
c.208+2T>C	p.?	DM	Splice	Imani (2019) Biosci Rep
c.522+3A>G		DM	Splice	Li (2004) Cell
c.619-27T>G		DM	Splice	Hirano (2015) PLoS One
c.619-1G>C		DM?	Splice	Feuillan (2011) J Clin Endocrinol Metab
c.900G>C	(p.Val300=)	DM	Splice	Carss (2017) Am J Hum Genet
c.123delA	p.(Gly42Glufs*11)	DM	Frameshift	Smaoui (2006) Invest Ophthalmol Vis Sci
c.734_744del11	p.(Glu245Glyfs*18)	DM	Frameshift	Maria (2016) Sci Rep
c.955_957delGAA	p.(Glu319del)	DM	Indel inframe	
c.54dupC	p.(Ala19Argfs*14)	DM	Frameshift	Feuillan (2011) J Clin Endocrinol Metab
c.966dupT	p.(Ala323Cysfs*57)	DM	Frameshift	Al-Hamed (2014) Cilia
c.263_271delTACGAGGCCinsGCTCTTA	p.(Leu88Cysfs*3)	DM	Frameshift	Li (2004) Cell
ex. 6-7, c.387-?_618+?del232		DM	CNV	Deveault (2011) Hum Mutat
ex. 9-12		DM	CNV	Nishimura (2005) Am J Hum Genet
incl. ex. 8-12		DM	CNV	Lindstrand (2016) Am J Hum Genet

Supplementary Table S7. List of known *BBS5* pathogenic variants according to the HGMD pro (2021.1). The *BBS5* reference sequence used is NM_152384.3. CNV: Copy Number Variant, DM: disease-causing mutation, “DM?”: likely disease-causing mutation.

Family	#Patient	Sex	Ethnicity	Cons.	Disease	Gene	HGVS nomenclature	Type	Zygosity	RP	OB	PD	HG	RD	ID	DD	Other features	Reference	PMID
1	1	F	European	N	BBS	*BBS1,*<br>*BBS5*	*BBS1*: c.1169TG (p.Met390Arg), del ex. 10-17,<br>*BBS5*: c.551A>G (p.Asn184Ser) (modifier)	Missense, CNV,<br>Missense	Het	Y	Y	Y		N	Y	N	Scoliosis, otitis media	Li (2004) Cell<br>Lindstrand (2016) AJHG	15137946<br>27486776
2	1		European		BBS	*BBS5*	c.620G>A, p.(Arg207His)	Missense	Het									Li (2004) Cell	15137946
3	2		New<br>founland		BBS	*BBS5*	c.522+3A>G	Splice										Li (2004) Cell<br>Hjortshoj (2008) AJMG A	15137946<br>18203199
			New<br>founland		BBS	*BBS5*	c.522+3A>G	Splice										Li (2004) Cell<br>Hjortshoj (2008) AJMG A	15137946<br>18203199
4	1		Turkey		BBS	*BBS5*	c.263_271delins, p.(Leu88Cysfs*3)	Frameshift	Hom									Li (2004) Cell	15137946
5	1		Kurdish		BBS	*BBS5*	c.176G>A, p.(Trp59*)		Hom									Li (2004) Cell<br>Hjortshoj (2008) AJMG A	15137946<br>18203199
6	3		Saudi<br>Arabia		BBS	*BBS5*	c.181T>A/G, p.(Leu142*)		Hom									Li (2004) Cell<br>Hjortshoj (2008) AJMG A	15137946<br>18203199
			Saudi<br>Arabia		BBS	*BBS5*	c.181T>A/G, p.(Leu142*)		Hom									Li (2004) Cell<br>Hjortshoj (2008) AJMG A	15137946<br>18203199
			Saudi<br>Arabia		BBS	*BBS5*	c.181T>A/G, p.(Leu142*)		Hom									Li (2004) Cell<br>Hjortshoj (2008) AJMG A	15137946<br>18203199
7	1		Turkey	Y	BBS	*BBS5*	Del ex. 9-12	CNV	Hom		Y	Y		Y				Nishimura (2005) AJHG	16380913
8	1	M	Tunisian	Y	BBS	*BBS5*	c.123delA, p.(Gly42Glufs*11)	Frameshift	Hom									Smaoui (2006) IOVS	16877420
9	4	M	Somalia	N	BBS	*BBS5*	c.214G>A, p.(Gly72Ser)	Missense	Hom									Hjortshoj (2008) AJMG A	18203199
		M	Somalia	N	BBS	*BBS5*	c.214G>A, p.(Gly72Ser)	Missense	Hom									Hjortshoj (2008) AJMG A	18203199
		M	Somalia	N	BBS	*BBS5*	c.214G>A, p.(Gly72Ser)	Missense	Hom									Hjortshoj (2008) AJMG A	18203199
		M	Somalia	N	BBS	*BBS5*	c.214G>A, p.(Gly72Ser)	Missense	Hom									Hjortshoj (2008) AJMG A	18203199
10	1	M	Sri Lanka	N	BBS	*BBS5*	c.547A>G, p.(Thr183Ala)	Missense	Hom									Hjortshoj (2008) AJMG A	18203199
11	2		North<br>Africa		BBS	*BBS5*	c.1A>T, p.(Met1?)	Start loss	Hom									Muller (2010) Hum Genet	20177705
			North<br>Africa		BBS	*BBS5*	c.1A>T, p.(Met1?)	Start loss	Hom									Muller (2010) Hum Genet	20177705
12	2		North<br>Africa		BBS	*BBS5*	c.166A>G, p.(Arg56Gly)	Missense	Hom									Muller (2010) Hum Genet	20177705
			North<br>Africa		BBS	*BBS5*	c.166A>G, p.(Arg56Gly)	Missense	Hom									Muller (2010) Hum Genet	20177705
13	5		Algeria		BBS	*BBS5*	c.123delA, p.(Lys41fs*52)	Frameshift	Hom									Muller (2010) Hum Genet	20177705
			Algeria		BBS	*BBS5*	c.123delA, p.(Lys41fs*52)	Frameshift	Hom									Muller (2010) Hum Genet	20177705
			Algeria		BBS	*BBS5*	c.123delA, p.(Lys41fs*52)	Frameshift	Hom									Muller (2010) Hum Genet	20177705
			Algeria		BBS	*BBS5*	c.123delA, p.(Lys41fs*52)	Frameshift	Hom									Muller (2010) Hum Genet	20177705
			Algeria		BBS	*BBS5*	c.123delA, p.(Lys41fs*52)	Frameshift	Hom									Muller (2010) Hum Genet	20177705
14	1		Pakistan	Y	BBS	*BBS5*	c.2T>A, p.(Met1?)	Start loss	Hom	Y	Y	Y	Y	N				Harville (2010) JMG	19797195
15	1		Pakistan	Y	BBS	*BBS5*	c.2T>A, p.(Met1?)	Start loss	Hom	Y	N	Y	Y	N				Harville (2010) JMG	19797195
16	1		Tunisian		BBS	*BBS5*	c.149T>G, p.(Leu50Arg)	Missense	Hom									Chen (2011) IOVS	21642631
17	1		European		BBS	*BBS5*	c.619–1G>C	Splice	Hom									Chen (2011) IOVS	21642631
18	1				BBS	*BBS5*	c.889G>A, p.(Asp297Asn)	Missense	Het		Y						Hyperleptinemia	Feuillan (2011) JCEM	21209035
19	1				BBS	*BBS5*	c.619-1G>C	Splice	Het		Y						Hyperleptinemia	Feuillan (2011) JCEM	21209035
20	1				BBS	*BBS5*	c.54dupC (p.(Ala19Argfs*14)), c.619-1G>C	Frameshift,<br>splice	Comp. Het		Y						Hyperleptinemia	Feuillan (2011) JCEM	21209035
21	1	F	Jordanian	Y	BBS	*BBS5*	Del ex. 6-7	CNV	Hom	Y	Y	Y	Y	N	Y	Y	Asthma, dislipidemia, teeth crowding, sleep apnea	Deveault (2011) Hum Mutat	21344540
22	1	M	Somalia	Y	BBS	*BBS5*	c.214G>A, p.(Gly72Ser)	Missense	Hom	Y	Y	Y		N	N	Y	Liver structure anomaly, psychosis, teeth crowding, small genitalia	Deveault (2011) Hum Mutat	21344540
23	1		Arabe	Y	BBS	*BBS5,*<br>*BBS1,*<br>*BBS9*	BBS5: c.158C>T, p.(Thr53Ile)<br>BBS1: c.1684G>A, p.(Asp562Asn)<br>BBS9: c.1693+102G>A	Missense<br>Missense<br>Splice?	Hom	Y	Y	Y	Y	N	Y	N	Atopy, Typical facies	Abu-Safieh (2012) EJHG	22353939
24	1		Arabe	Y	BBS	*BBS1,*<br>*BBS5,*<br>*BBS4,*<br>*CEP290*	BBS1:c.124+1G>A (p.(Leu43Glyfs*44)), c.951+58C>T<br>BBS5: c.132T>G, p.(Asn44Lys) (modifier)<br>BBS4: c.221-37G>A<br>CEP290: c.6271-113T>C	Splice<br>Missense	Het	Y	Y	Y	Y	N	Y	N	Typical facies	Abu-Safieh (2010) JMG<br>Abu-Safieh (2012) EJHG	19858128<br>22353939
25	1		France	N	BBS	*BBS5*	c.413G>A, (p.(Arg138His))	Missense		Y	N	Y	Y	N	Y	N	Atopy, typical facies	Redin (2012) JMG	22773737
26	1				RP	*BBS5*	c.148C>A, p.(Leu50Ile)	Missense	Hom	Y	N	N	N	N	N	N		Wang (2014) Hum Genet	24154662
27	3		Saudi<br>Arabia	Y	BBS	*BBS5*	c.966dupT, p.(Ala323Cysfs*57)	Frameshift	Hom	Y	Y	Y	N	Y	Y	N	Hydronephrosis, enlarged fatty liver, typical facies	Al-Hamed (2014) Cilia<br>Shaheen (2016) Genome Biol	24559376<br>27894351

			Saudi Arabia	Y	BBS	*BBS5*	c.966dupT, p.(Ala323Cysfs*57)	Frameshift	Hom	N	Y	Y	N	Y	Y	N	Fatty liver, typical facies, atopy	Al-Hamed (2014) Cilia<br>Shaheen (2016) Genome Biol	24559376<br>27894351
			Saudi Arabia	Y	BBS	*BBS5*	c.966dupT, p.(Ala323Cysfs*57)	Frameshift	Hom	N	Y	N	N	Y	Y	N	Hydronephrosis, fatty liver, typical facies, atopy	Al-Hamed (2014) Cilia<br>Shaheen (2016) Genome Biol	24559376<br>27894351
28	2	M	Japenese	N	BBS	*BBS5*	c.265C>T (p.Arg89*), c.619-27T>G	Nonsense, Splice	Comp. Het	N	Y	N	Y	N	Y	N	Esophageal, gastric and rectal varices, liver fibrosis	Hirano (2015) PLoS One	26325687
		M	Japenese	N	BBS	*BBS5*	c.265C>T (p.Arg89*), c.619-27T>G	Nonsense, Splice	Comp. Het	N	Y	Y	Y	N	Y	N		Hirano (2015) PLoS One	26325687
29	1	M	European	N	BBS	*BBS1, BBS5*	BBS1: c.1645G>T (p.Glu549*), del ex. 1-11,<br>*BBS5*: c.584A>G (p.Asp195Gly) (modifier)	CNV, frameshift<br>Missense	Het	Y	Y	Y		Y	Y	Y	Scoliosis, otitis media	Lindstrand (2016) AJHG	27486776
30	1	M	European	N	BBS	*BBS10, BBS5*	*BBS10*:c.2119_2120delGT (p.Val707*) hom<br>*BBS5*: incl. ex. 8-12	CNV<br>CNV	Het	Y	Y	Y		Y	Y		Seizures, sleep apnea	Lindstrand (2016) AJHG	27486776
31	1			Y	BBS	*BBS5*	c.966dupT, p.(Ala323Cysfs*57)	Frameshift	Hom	Y	Y	Y	Y	Y	Y	N	Grade 4 bilateral hydronephrosis, small trabeculated bladder	Shaheen (2016) Genome Biol	27894351
32	1			Y	BBS	*BBS5*	c.966dupT, p.(Ala323Cysfs*57)	Frameshift	Hom	Y	Y	N	N	N	N	N	Brachydactyly, atopy, asthma, hypogenitalism, typical facies	Shaheen (2016) Genome Biol	27894351
33	2			Y	BBS	*BBS5*	c.966dupT, p.(Ala323Cysfs*57)	Frameshift	Hom	N	Y	N	N	N	Y	N	Acanthosis nigricans, macrocephaly, poor vision	Shaheen (2016) Genome Biol	27894351
				Y	BBS	*BBS5*	c.966dupT, p.(Ala323Cysfs*57)	Frameshift	Hom	N	Y	N	N	N	Y	N	Acanthosis nigricans, macrocephaly, poor vision	Shaheen (2016) Genome Biol	27894351
34	1			Y	BBS	*BBS5*	c.966dupT, p.(Ala323Cysfs*57)	Frameshift	Hom	N	Y	N	Y	N	Y	N		Shaheen (2016) Genome Biol	27894351
35	2	M	Pakistani	Y	BBS	*BBS5*	c.734_744del11, p.(Glu245Glyfs*18)	Frameshift	Hom	Y	N	Y	Y	Y	Y	N	Hypodontia, syndactyly, brachydactyly, ataxia, speech disability, gall bladder calculi, mild spleno- and hepatomegaly, elevated liver enzymes, abnormally high cholesterol level	Maria (2016) Sci Rep	27708425
		M	Pakistani	Y	BBS	*BBS5*	c.734_744del11, p.(Glu245Glyfs*18)	Frameshift	Hom	Y	N	N	Y	Y	Y	N	Speech disability	Maria (2016) Sci Rep	27708425
36	1	F	Pakistani	Y	BBS	*BBS5*	c.734_744del11, p.(Glu245Glyfs*18)	Frameshift	Hom	Y	N	Y	Y	N	N	Y	Irregular menstruation, low progesterone levels, diabetes, borderline hepatomegaly with fatty infiltration, abnormally high cholesterol level, elevated liver enzymes	Maria (2016) Sci Rep	27708425
37	2				BBS	*BBS5*	c.790G>A, p.(Gly264Arg)	Missense	Hom	N	Y	N	N	N	N		Achromatopsia in childhood	Weisschuh (2016) PLoS One	26766544
					BBS	*BBS5*	c.790G>A, p.(Gly264Arg)	Missense	Hom	N	Y	N	N	N	N		Achromatopsia in childhood	Weisschuh (2016) PLoS One	26766544
38	2			Y		*BBS5*	c.532G>A, p.(Gly178Arg)	Missense	Hom	Y	Y	N	N	N	N	Y		Shaheen (2016) Genome Biol	27894351
				Y		*BBS5*	c.532G>A, p.(Gly178Arg)	Missense	Hom	N	Y	N	N	N	N	Y	Nystagmus, poor vision, sleep apnea, delayed myelination	Shaheen (2016) Genome Biol	27894351
39	1			Y		*BBS5*	c.1A>T, p.?	Start loss	Hom	N	Y	Y	N	Y	N	N		Shaheen (2016) Genome Biol	27894351
40	1				BBS	*BBS5*	c.955_957delGAA, p.(Glu319del)	Indel inframe	Hom			Y				Y	Small testis, CHD, ADHD, dysmorphic facies, echogenic kidneys, cleft lip, normal brain MRI, echogenic kidney	Patel (2016) Genet Med<br>Shaheen (2016) Genome Biol	26355662<br>27894351
41	1	F			CD	*BBS5*	c.900G>C, p.(Val300=)	Splice	Het	Y	N	N	N	N	N	N		Carss (2017) AJHG	28041643
42	1	F			CD	*BBS5*	c.412C>T, p.(Arg138Cys)	Missense	Het	Y	N	N	N	N	N	N		Carss (2017) AJHG	28041643
43	1	F			CD	*BBS5*	c.551A>G, p.(Asn184Ser)	Missense	Het	Y	N	N	N	N	N	N		Carss (2017) AJHG	28041643
44	1	M	Indian	N	BBS	*BBS5*	c.425T>G, p.(Leu142*)	Nonsense	Hom									Chandrasekar (2018) IJMR	29806606
45	2	M	Iranian	N	BBS	*BBS5*	c.208+2T>C	Splice	Hom	Y	Y	N	Y	N	N	N	Speech disability, dental anomaly, attenuated arterioles, pale optic disc, night blindness	Imani (2019) Biosci Rep	30850397
		F	Iranian	N	BBS	*BBS5*	c.208+2T>C	Splice	Hom	Y	Y	N	N	Y	N	N	Posterior subcapsular cataract, digestion problem, attenuated arterioles, pale optic disc, bone spicules, macular atrophy, night blindness	Imani (2019) Biosci Rep	30850397

Supplementary Table S8. List of known *BBS5* cases with pathogenic variants organized as case according to the HGMD pro (2021.1). The *BBS5* reference sequence used is NM_152384.3. When multiple gene are indicated in the gene column, the first one is considered as the primary driver gene. CNV: Copy Number Variant, Hom: homozygous, Het: heterozygous, Comp. het: compound heterozygous, CD: Cone dystrophy, RP: Retinitis pigmentosa, Ob: Obesity, PD: Polydactyly,HG: Hypogonadism, RD: Renal disease, ID: Intellectual disability DD: Developmental delay, CHD: Congenital heart disease, ADHD: Attention deficit hyperactivity disorder.

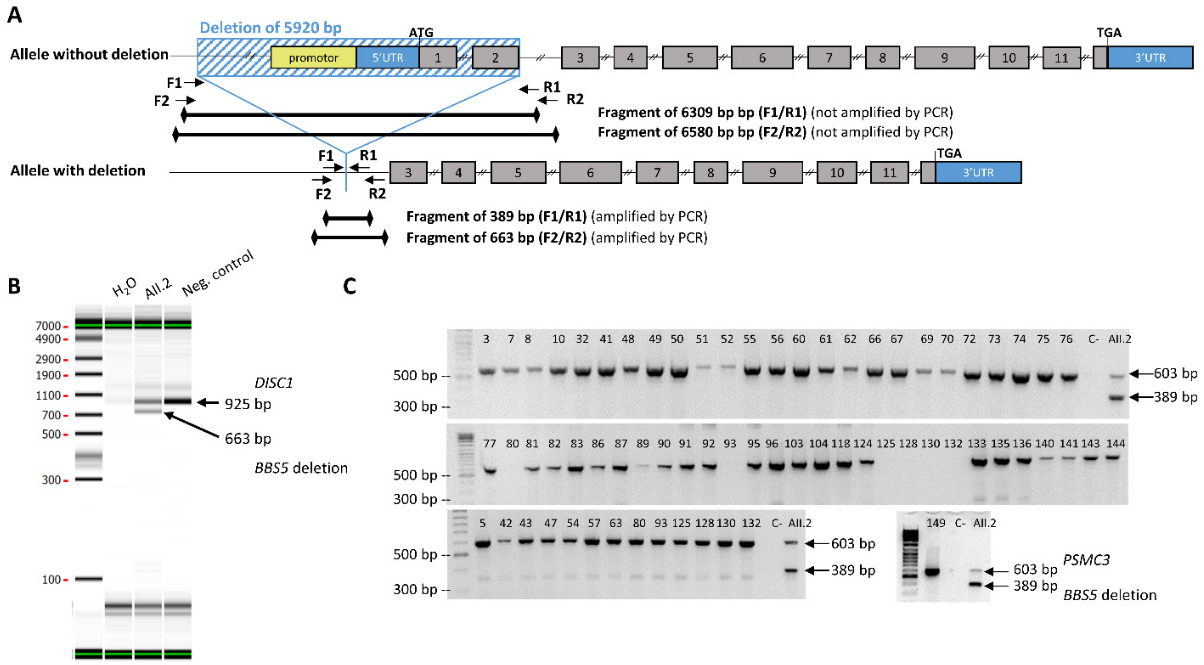

Supplementary Figure S1. Cohort screening for the *BBS5* deletion. **A.** Presentation of the primers positions on *BBS5* for Duplex 1 and 2 designed for the detection of the deletion of exons 1 and 2. **B.** Example of PCR profiles for Duplex 2 on a LabChip GX (Perkin Elmer) for a rapid detection of the *BBS5* deletion (see for primers details). Normal samples harbour a single band (*DISC1*) whereas homozygous or heterozygous carriers for the tandem duplication show an additional band specific for the duplication breakpoint. **C.** Results of the Duplex 1 PCR performed on 62 BBS negative samples out of 284. Except for the index case, the deletion was not found in any other patient.

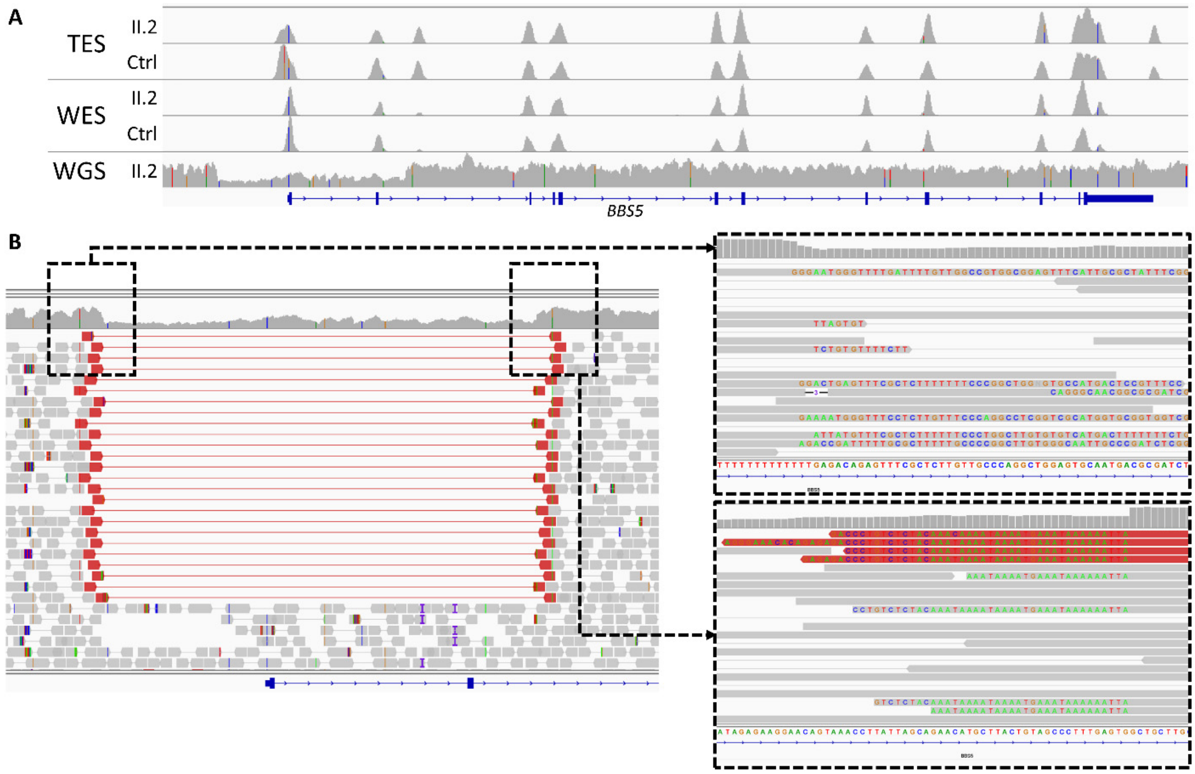

Supplementary Figure S2. *BBS5* large heterozygous deletion. **A.** Comparison of the *BBS5* depth of coverage for patient II.2 and a control using either the BBS gene panel (TES), whole exome sequencing (WES) or whole genome sequencing (WGS) using IGV. **B.** Highlight of the split reads on each side of the deletion displaying the breakpoint of the 5,918 bp heterozygous deletion of exons 1 and 2 of *BBS5*.

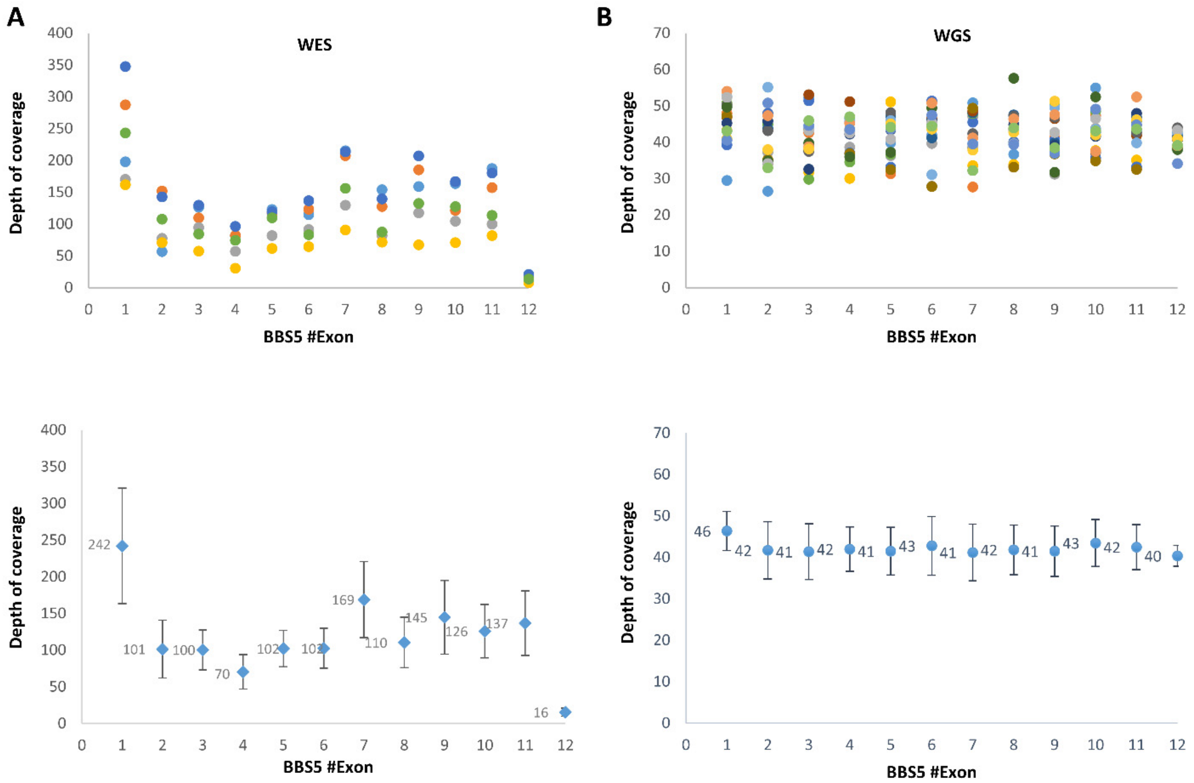

Supplementary Figure S3. Distribution of the depth of coverage (upper panels) and the mean and its standard deviation (lower panels) for each exon of *BBS5* for 6 WES from the same sequencing run (A.) and for 18 WGS (B.). Depth of coverage was computed using the DepthOfCoverage command from GenomeAnalysisTK-3.4-46 and the GRCh37 reference coordinates for the *BBS5* gene (NM_152384.3).

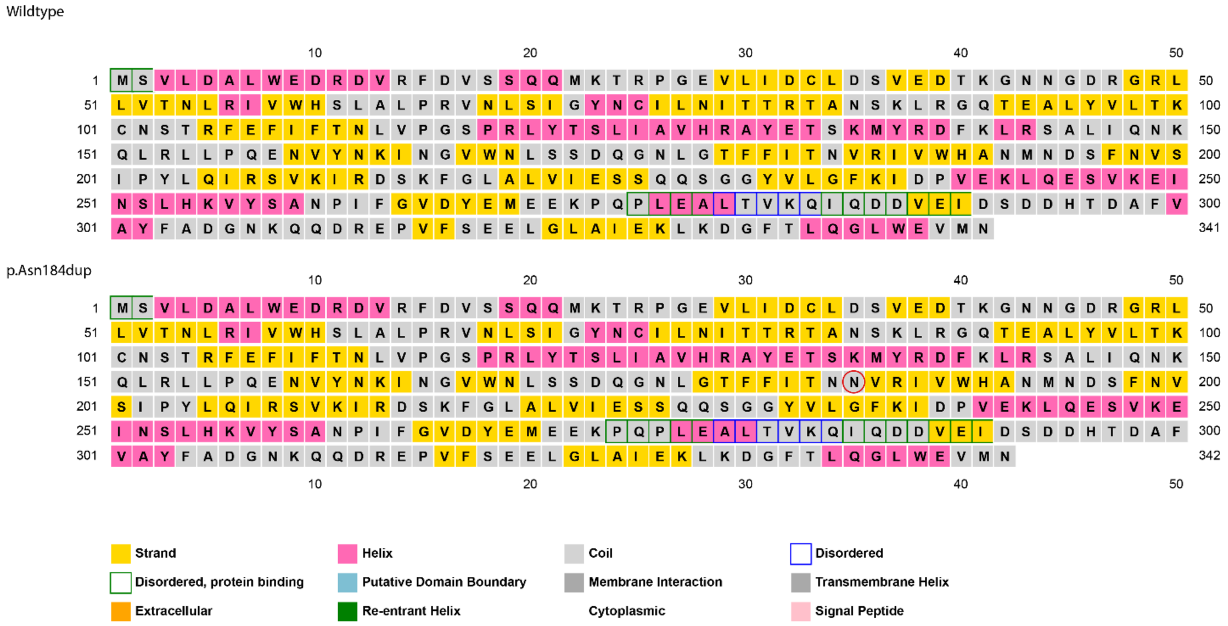

Supplementary Figure S4. Secondary structure prediction. PSIPRED [25] was applied to assess whether the p.Asn184dup (panel below, duplication is highlighted by a red circle) might affect the secondary structure of BBS5.

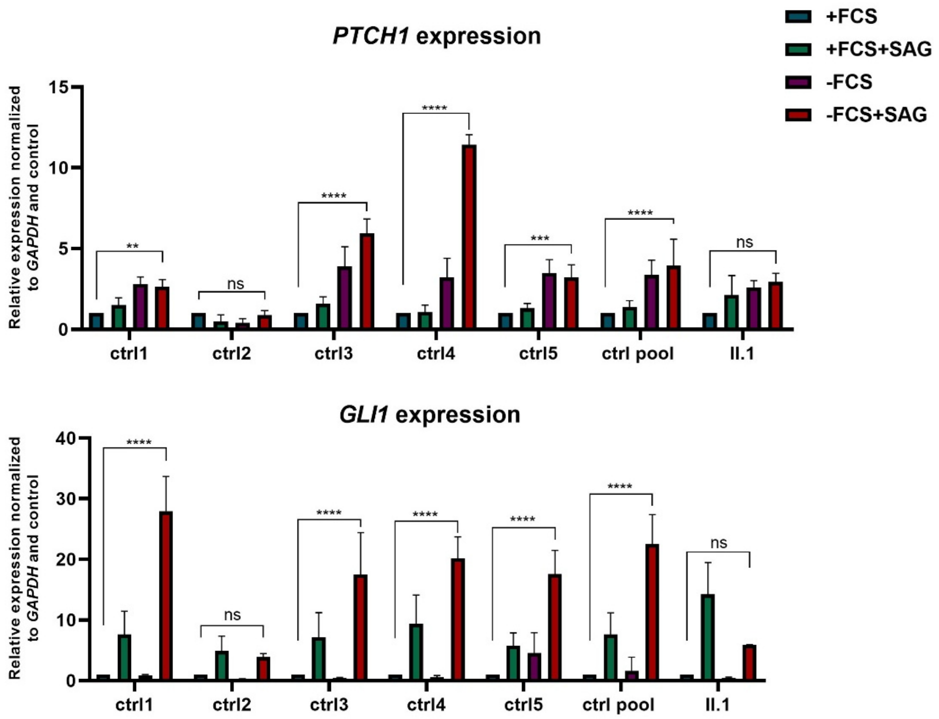

Supplementary Figure S5. Assessment of Hh-signal transduction by quantification of the induction of two Hh target genes *PTCH1* and *GLI1* on Hh-stimulated cells from controls and the patient in four different conditions, with and without inducing ciliogenesis (+/- FCS) and Hh activation (+/- SAG). The expression of both genes is normally induced in controls except in control 2, whereas no induction is found in the patient test. The expression data performed with biological triplicates were normalized to the reference gene *GAPDH* mRNA expression data, and the values were presented as relative expression levels +- SEM). *p*-values and significance were determined using 2-way ANOVA test (Tukey's multiple comparison test). ns: non significant, **p<0.01 ****p<0.0001.

SUPPLEMENTARY REFERENCES

1. Thorvaldsdóttir, H.; Robinson, J.T.; Mesirov, J.P. Integrative Genomics Viewer (IGV): High-Performance Genomics Data Visualization and Exploration. *Briefings in Bioinformatics* **2012**, *14*, 178–192, doi:10.1093/bib/bbs017.
2. Li, H.; Durbin, R. Fast and Accurate Short Read Alignment with Burrows–Wheeler Transform. *Bioinformatics* **2009**, *25*, 1754–1760, doi:10.1093/bioinformatics/btp324.
3. DePristo, M.A.; Banks, E.; Poplin, R.; Garimella, K.V.; Maguire, J.R.; Hartl, C.; Philippakis, A.A.; del Angel, G.; Rivas, M.A.; Hanna, M.; et al. A Framework for Variation Discovery and Genotyping Using Next-Generation DNA Sequencing Data. *Nature Genetics* **2011**, *43*, 491–498, doi:10.1038/ng.806.
4. Geoffroy, V.; Pizot, C.; Redin, C.; Piton, A.; Vasli, N.; Stoetzel, C.; Blavier, A.; Laporte, J.; Muller, J. VaRank: A Simple and Powerful Tool for Ranking Genetic Variants. *PeerJ* **2015**, *3*, e796, doi:10.7717/peerj.796.
5. The 1000 Genomes Project Consortium; Gibbs, R.A.; Boerwinkle, E.; Doddapaneni, H.; Han, Y.; Korchina, V.; Kovar, C.; Lee, S.; Muzny, D.; Reid, J.G.; et al. A Global Reference for Human Genetic Variation. *Nature* **2015**, *526*, 68–74, doi:10.1038/nature15393.
6. Lek, M.; Karczewski, K.J.; Minikel, E.V.; Samocha, K.E.; Banks, E.; Fennell, T.; O'Donnell-Luria, A.H.; Ware, J.S.; Hill, A.J.; Cummings, B.B.; et al. Analysis of Protein-Coding Genetic Variation in 60,706 Humans. *Nature* **2016**, *536*, 285-+, doi:10.1038/nature19057.
7. MacDonald, J.R.; Ziman, R.; Yuen, R.K.C.; Feuk, L.; Scherer, S.W. The Database of Genomic Variants: A Curated Collection of Structural Variation in the Human Genome. *Nucleic Acids Res.* **2014**, *42*, D986-992, doi:10.1093/nar/gkt958.
8. Nevers, Y.; Prasad, M.K.; Poidevin, L.; Chennen, K.; Allot, A.; Kress, A.; Ripp, R.; Thompson, J.D.; Dollfus, H.; Poch, O.; et al. Insights into Ciliary Genes and Evolution from Multi-Level Phylogenetic Profiling. *Molecular Biology and Evolution* **2017**, *34*, 2016–2034, doi:10.1093/molbev/msx146.
9. Thung, D.T.; de Ligt, J.; Vissers, L.E.; Steehouwer, M.; Kroon, M.; de Vries, P.; Slagboom, E.P.; Ye, K.; Veltman, J.A.; Hehir-Kwa, J.Y. Mobster: Accurate Detection of Mobile Element Insertions in next Generation Sequencing Data. *Genome Biology* **2014**, *15*, 488, doi:10.1186/s13059-014-0488-x.
10. Geoffroy, V.; Herenger, Y.; Kress, A.; Stoetzel, C.; Piton, A.; Dollfus, H.; Muller, J. AnnotSV: An Integrated Tool for Structural Variations Annotation. *Bioinformatics* **2018**, doi:10.1093/bioinformatics/bty304.
11. Talevich, E.; Shain, A.H.; Botton, T.; Bastian, B.C. CNVkit: Genome-Wide Copy Number Detection and Visualization from Targeted DNA Sequencing. *PLOS Computational Biology* **2016**, *12*, e1004873, doi:10.1371/journal.pcbi.1004873.
12. Suvakov, M.; Panda, A.; Diesh, C.; Holmes, I.; Abyzov, A. CNVpytor: A Tool for Copy Number Variation Detection and Analysis from Read Depth and Allele Imbalance in Whole-Genome Sequencing. *Gigascience* **2021**, *10*, giab074, doi:10.1093/gigascience/giab074.
13. Rausch, T.; Zichner, T.; Schlattl, A.; Stütz, A.M.; Benes, V.; Korbel, J.O. DELLY: Structural Variant Discovery by Integrated Paired-End and Split-Read Analysis. *Bioinformatics* **2012**, *28*, i333–i339, doi:10.1093/bioinformatics/bts378.
14. Layer, R.M.; Chiang, C.; Quinlan, A.R.; Hall, I.M. LUMPY: A Probabilistic Framework for Structural Variant Discovery. *Genome Biol* **2014**, *15*, R84, doi:10.1186/gb-2014-15-6-r84.
15. Backenroth, D.; Homsy, J.; Murillo, L.R.; Glessner, J.; Lin, E.; Brueckner, M.; Lifton, R.; Goldmuntz, E.; Chung, W.K.; Shen, Y.F. CANOES: Detecting Rare Copy Number Variants from Whole Exome Sequencing Data. *Nucleic Acids Res* **2014**, *42*, doi:10.1093/nar/gku345.

16. Chen, X.; Schulz-Trieglaff, O.; Shaw, R.; Barnes, B.; Schlesinger, F.; Källberg, M.; Cox, A.J.; Kruglyak, S.; Saunders, C.T. Manta: Rapid Detection of Structural Variants and Indels for Germline and Cancer Sequencing Applications. *Bioinformatics* **2016**, *32*, 1220–1222, doi:10.1093/bioinformatics/btv710.
17. Bartenhagen, C.; Dugas, M. Robust and Exact Structural Variation Detection with Paired-End and Soft-Clipped Alignments: SoftSV Compared with Eight Algorithms. *Briefings in Bioinformatics* **2016**, *17*, 51–62, doi:10.1093/bib/bbv028.
18. Huerta-Cepas, J.; Szklarczyk, D.; Forslund, K.; Cook, H.; Heller, D.; Walter, M.C.; Rattei, T.; Mende, D.R.; Sunagawa, S.; Kuhn, M.; et al. EggNOG 4.5: A Hierarchical Orthology Framework with Improved Functional Annotations for Eukaryotic, Prokaryotic and Viral Sequences. *Nucleic Acids Research* **2015**, *44*, D286–D293, doi:10.1093/nar/gkv1248.
19. Singh, S.K.; Gui, M.; Koh, F.; Yip, M.C.; Brown, A. Structure and Activation Mechanism of the BBSome Membrane Protein Trafficking Complex. *Elife* **2020**, *9*, doi:10.7554/eLife.53322.
20. Waterhouse, A.M.; Procter, J.B.; Martin, D.M.A.; Clamp, M.; Barton, G.J. Jalview Version 2--a Multiple Sequence Alignment Editor and Analysis Workbench. *Bioinformatics* **2009**, *25*, 1189–1191, doi:10.1093/bioinformatics/btp033.
21. Schrödinger, LLC The PyMOL Molecular Graphics System, Version 1.8 2015.
22. Hu, J.; Ng, P.C. SIFT Indel: Predictions for the Functional Effects of Amino Acid Insertions/Deletions in Proteins. *PLoS One* **2013**, *8*, e77940, doi:10.1371/journal.pone.0077940.
23. Zhao, H.; Yang, Y.; Lin, H.; Zhang, X.; Mort, M.; Cooper, D.N.; Liu, Y.; Zhou, Y. DDIG-in: Discriminating between Disease-Associated and Neutral Non-Frameshifting Micro-Indels. *Genome Biology* **2013**, *14*, R23, doi:10.1186/gb-2013-14-3-r23.
24. Choi, Y.; Chan, A.P. PROVEAN Web Server: A Tool to Predict the Functional Effect of Amino Acid Substitutions and Indels. *Bioinformatics* **2015**, *31*, 2745–2747, doi:10.1093/bioinformatics/btv195.
25. Buchan, D.W.A.; Jones, D.T. The PSIPRED Protein Analysis Workbench: 20 Years On. *Nucleic Acids Research* **2019**, *47*, W402–W407, doi:10.1093/nar/gkz297.
